# Enhanced Eradication of *Enterococcus faecalis* Biofilms by Quaternized Chitosan-Coated Upconversion Nanoparticles for Photodynamic Therapy in Persistent Endodontic Infections

**DOI:** 10.3389/fmicb.2022.909492

**Published:** 2022-05-31

**Authors:** Bin Zong, Xue Li, Quanchen Xu, Danyang Wang, Pengyu Gao, Qihui Zhou

**Affiliations:** ^1^Department of Stomatology, The Affiliated Hospital of Qingdao University, Qingdao University, Qingdao, China; ^2^School of Stomatology, Qingdao University, Qingdao, China; ^3^Institute for Translational Medicine, The Affiliated Hospital of Qingdao University, Qingdao University, Qingdao, China

**Keywords:** upconversion nanoparticles, quaternized chitosan, photodynamic therapy, reactive oxygen species, anti-biofilm

## Abstract

Due to the persistent presence of *Enterococcus faecalis* biofilms in apical root canals, persistent endodontic infections (PEIs) have always been an intractable disease to solve. The conventional root canal disinfectants (e.g., calcium hydroxide, chlorhexidine) are arduous to scavenge the stubborn infection. With the progress of nanomedicine in the biomedical field, antimicrobial photodynamic therapy (aPDT) is emerging as a prospective anti-infective therapy for PEIs. Herein, quaternized chitosan (QCh) modified upconversion nanoparticles (UCNP)@SiO_2_/methylene blue (MB) are developed with enhanced antibacterial/biofilm performance for aPDT in PEIs. QCh is coated on the UCNP@SiO_2_/MB by testing the changes in diameter, chemical functional group, and charge. Interestingly, QCh also increases the conversion efficiency of UCNP to generate more reactive oxygen species (ROS). Furthermore, the prepared UCNP@SiO_2_/MB@QCh exhibits highly effective antibacterial activity against free *E. faecalis* and related biofilm *in vitro* and extracted teeth. Importantly, the additional QCh with positive charges enhance UCNP@SiO_2_/MB@QCh contact with *E. faecalis* (negative charges) through electrostatic interaction. Then, UCNP@SiO_2_/MB@QCh could stick close to the *E. faecalis* and generate ROS under the irradiation by a 980 nm laser. The *in vitro* cellular test shows that UCNP@SiO_2_/MB@QCh has acceptable cytocompatibility. Thus, UCNP@SiO_2_/MB@QCh could offer a novel strategy for the potential aPDT clinical applications in the treatment of PEIs.

## Graphical Abstract

Schematic diagram of the preparation of UCNP@SiO_2_/MB@QCh and the following antibacterial effect. 
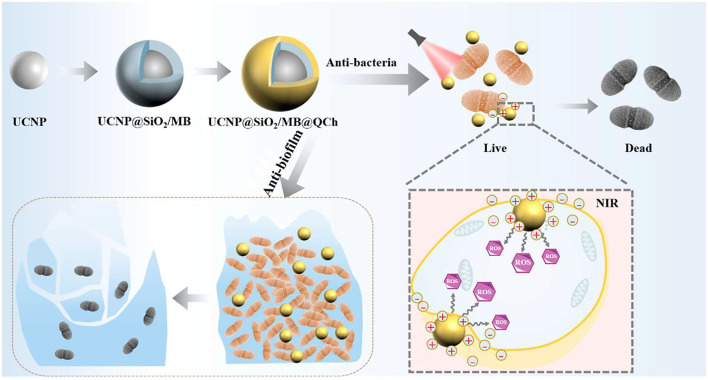

## Introduction

Apical periodontitis (AP) is a destructive inflammatory disease of the apical periodontium (periodontal ligament, cementum, and bone), which resulted from persistent endodontic infections (PEIs) (Bordagaray et al., 2021). A new systematic review study from 2012 to 2020 has revealed that there is a notably increased prevalence of PEIs in adults compared with data from 2012 (6.3 vs. 5.4%) (Jakovljevic et al., 2020). Considering the stubbornly high prevalence, practical and effective treatment for PEIs is urgent (Wang et al., 2020a).

*Enterococcus faecalis* (*E. faecalis*) is a crucial etiologic pathogen of PEIs, and the pathogenicity of *E. faecalis* in biofilms is 100–1,000 times higher than in planktonic states (Bowen et al., 2018). However, relevant research reported that more than 40% of *E. faecalis* clinical isolates could form biofilms, so that AP becomes refractory (Feng et al., 2021; Ji et al., 2021). In the territory of EIs, the methods applied to eliminate bacteria from the root canal system mainly incorporate calcium hydroxide and antibiotics such as chlorhexidine. However, these medicaments are often associated with unsatisfied curative effects and inevitable adverse effects, such as discoloration of the tongue and teeth caused by chlorhexidine (Lei et al., 2016). Hence, developing a high-performance antibacterial biomaterial for eradicating *E. faecalis* biofilm is critical to the treatment of PEIs.

With the progress of nanomedicine in the biomedical field (Wang et al., 2021b; Qi et al., 2022; Yan et al., 2022), antimicrobial photodynamic therapy (aPDT) has attracted increasing attention as a promising means to scavenge pathogenic microorganisms from infectious lesions (de Annunzio et al., 2018; Zhang et al., 2020). Generally, the bactericidal mechanism of aPDT relies on the reactive oxygen species (ROS), which is produced by the photosensitizers (PSs) and oxygen under the irradiation of light with a particular wavelength (Hu et al., 2018; Zhao et al., 2021). Nevertheless, commonly clinically used PS, such as methylene blue (MB), have some deficiencies, including poor water-solubility, uncontrollable drug-release profile, poor target selectivity, and low extinction coefficient (Yin et al., 2015). In addition, their excitation light, such as ultraviolet light, causes tissue damage and visible light possesses inadequate tissue-penetration ability. To overcome the abovementioned limitations, lanthanide-doped upconversion nanoparticles (UCNPs) could serve as PSs or PS carriers owing to the merits of UCNPs (e.g., high photostability, low cytotoxicity, and sharp emission lines) (Lu et al., 2016; Lee et al., 2020). UCNPs could be able to absorb near-infrared (NIR) light and convert it into high-energy photons. The NIR light ranging from 700 to 1,100 nm had advantages of deeper tissue penetration and lower autofluorescence and could reduce side effects such as phototoxicity and photodamage. To enhance the efficiency of upconversion luminescence (UCL), a silica layer was applied to carry PSs on the surface of UCNPs, such as dense silica and mesoporous silica nanoparticles (Liu et al., 2015; Bayir et al., 2018). For instance, Liu et al. verified the antibacterial effect of UCNP@SiO_2_/MB against *Staphylococcus aureus* and *Escherichia coli*. The death rates of these two bacteria were achieved at 37.56 ± 4.00% and 63.97 ± 5.26%, respectively (Liu et al., 2020). The antibacterial effect of UCNP@SiO_2_/MB was relatively low for Gram-negative bacteria, as the negatively charged nanoparticles were difficult to contact and interact with negatively charged bacteria rapidly and directly (Yue et al., 2018; Ahmed et al., 2020). Chitosan and its derivatives have positive charges. Chitosan has native characteristics, including biocompatibility and non-toxicity (Hao et al., 2021, 2022; Zheng et al., 2021). However, the relatively low solubility in physiological solutions restricts the use of chitosan. In contrast, the quaternized chitosan (QCh) has excellent water solubility. Meanwhile, it could enhance the conversion efficiency of UCNP. Hence, we chose QCh to modify UCNP, instead of chitosan. It is well-demonstrated that QCh with good biocompatibility and strong positive charges could identify, interact, and disturb the membrane of bacteria, which might increase the membrane permeability and lead them to death (Ao et al., 2020; Phuangkaew et al., 2022). Therefore, UCNP@SiO_2_/MB@QCh would offer a novel strategy to treat *E. faecalis* associated PEIs for potential clinical applications, which has not been reported.

In our study, we designed a novel triple-layered core-shell nanostructure UCNP@SiO_2_/MB@QCh to eradicate *E. faecalis* for the treatment of PEIs. The antibacterial effects of UCNP@SiO_2_/MB@QCh against free *E. faecalis* and related biofilms were demonstrated *in vitro* through aPDT. Besides, the model of *E. faecalis* biofilm on extracted teeth was established to investigate their antibacterial effects further. The antibacterial experiment results showed that the antibacterial performance of the developed nanomaterials holds great potential for the management of PEIs.

## Experimental Sections

### Chemicals and Materials

Rare earth oxides (Y_2_O_3_, Yb_2_O_3_, Tm_2_O_3_, and Ho_2_O_3_), oleic acid (OA), 1-octadecene (ODE), tetraethyl orthosilicate (TEOS), sodium fluoride (NaF), igepal co-520(NP-5), sodium oleate, and 1,3-diphenylisobenzofuran (DPBF) were purchased from Shanghai Aladdin Biochemical Technology Co., Ltd. Methylene blue (MB, Mw 373.9), chlorhexidine digluconate (CHX,19-21%), n-hexane, and (3-chloro-2-hydroxypropyl)trimethylammonium chloride were provided by Shanghai Macklin Biochemical Co., Ltd. Cyclohexane, formaldehyde aqueous solution, ethanol absolute, urea, ammonia solution, acetonitrile, KOH, LiOH^.^H_2_O, and concentrated hydrochloric acid were supplied from Sinopharm Chemical Reagent Co., Ltd. Chitosan powder (Mv = 300 kDa, deacetylation degree ≥ 90%) was obtained from Zhejiang Golden-Shell Pharmaceutical Co., Ltd., China. Brain heart infusion (BHI) was obtained from Solarbio Science & Technology Co., Ltd. (Beijing, China). The FilmTracer™ LIVE/DEAD^®^ Biofilm Viability Kit was purchased from Thermo Fisher Scientific.

### Synthesis of UCNP@SiO_2_/MB@QCh

#### Synthesis of UCNP (NaYF_4_@NaYF_4_:Yb/Ho/Tm@NaYF_4_)

Yttrium oleate-Y(oleate)_3_ and Ln(oleate)_3_ (Ln = Y,Yb,Ho,Tm) were prepared by a previously reported method, with a molar ratio of lanthanide ions as Y:Yb:Tm:Ho = 77.8:20:0.2:2 (Song et al., 2017). 1.0 mmol Y(oleate)_3_ and 10 mmol NaF (0.84 g) were added into 20 ml oleic acid (OA) and 1-octadecene (ODE) solution (v/v = 1:1). After removing the air from the reactor and using N_2_ to avoid the oxidation of OA, the temperature was set at 110°C to react for an hour. Then, the reactor was heated up to 340°C for 1.5 h. Furthermore, 4 ml liquid was taken out and 8 ml mixed solution of 0.4 mmol Ln (oleate) _3_, OA/ODE (v/v = 1:1) was added to react for 20 min. Next, a 4 ml mixture was taken out from the flask. Then, 0.4 mmol of Y(oleate)_3_ in 8 ml of an OA/ODE mixing solvent (v/v = 1:1) was injected to prepare the outer shell NaYF_4_.

After reacting for 20 min, the temperature of the mixture solution was reduced to 25°C. The mixture solution was divided and poured into two centrifuge tubes, and an equal amount of ethanol absolute was injected to static settlement for 30 min. Finally, the mixture was centrifuged and the sediment was dissolved into cyclohexane.

#### Synthesis of UCNP@SiO_2_/MB

To achieve a thin dense silica layer, UCNP@SiO_2_/MB was synthesized through a successful method (Liu et al., 2015). First, 40 ml cyclohexane and 2 ml igepal co-520(NP-5) were stirred for an hour. Then, 40 mg UCNPs were added to the flask and stirred violently for 3 h. Then, 280 μl ammonia solution and 900 μl methylene blue (4 mg/l) were injected dropwise, respectively. After mixing for 2 h, the combined solution of tetraethyl orthosilicate (TEOS) and cyclohexane was added slowly by a TJP-3A Syringe Pump Controller (LongerPump) for an hour. After 24 h, 10 ml formaldehyde aqueous solution was injected to mix for 30 min. Finally, the compound was centrifuged and the sediment was dissolved in double distilled water (DDW) using ultrasound.

#### Synthesis of UCNP@SiO_2_/MB@QCh

QCh was prepared by a reported procedure (You et al., 2016). First of all, the purified QCh was dissolved into DDW through ultrasound. Then, the liquid of QCh whose concentration was twice that of UCNP@SiO_2_/MB was added into the latter dropwise under ultrasound. The ultrasound procedure lasted for 30 min. After the abovementioned procedures, the mixture liquid was centrifuged for 10 min at 12,000 rpm. The products were washed with DDW twice and finally dispersed in DDW for future application.

### Characterizations

Transmission electron microscopy (TEM) was obtained by Mic JEM-1200EX (Japan) to confirm the size and morphology. The power X-ray diffraction (XRD) pattern was recorded on a Rigaku Ultima IV with Cu-Kα radiation (λ = 0.15418). The morphology of samples was detected using SEM (VEGA3, TESCAN, Czech) operated at an acceleration voltage of 10 kV. A Nicolet iN10 Fourier transform-infrared (FTIR) spectrometer (Thermo Fisher Scientific, Waltham, MA, USA) was utilized to measure the FTIR spectra of the samples over the range of 500–4,000 cm^−1^ at a scanning resolution of 2 cm^−1^ during 32 scans. The luminescence intensity was recorded on an FLS980 fluorescence spectrometer (Edinburgh Instruments Ltd., UK) under 980 nm laser excitation at 1.5 W/cm^2^ with a NIR laser device. The UV-Vis spectrum was collected on UV-Vis Spectrophotometer (UV-8000, Shanghai). The zeta potential (Z) and diameter distribution (DLS) of products was detected by dynamic light scattering on a Zetasizer Nano ZSE (UK). The 3D-images of bacteria biofilms were carried out on a confocal laser scanning microscope (CLSM, Leica TCS SP8, Germany).

### Detection of Reactive Oxygen Species (ROS)

1,3-diphenylisobenzofuran (DPBF) was adopted to detect the generation of ROS from MB, indicating the PDT effect of nanoparticles. It is well known that DPBF can especially react with ROS, resulting in a decreased absorption intensity at 410 nm (Zhang et al., 2018b; Sun et al., 2020). The greater the reduction in absorption, the more ROS is generated. In brief, DPBF was dispersed in acetonitrile. MB, UCNP@SiO_2_, UCNP@SiO_2_/MB, and UCNP@SiO_2_/MB@QCh were prepared in ethanol absolute. Then, 20 μl DPBF (4 mg/ml) was added into 1.98 ml ethanol absolute containing the abovementioned materials, respectively. Moreover, 20 μl DPBF was resolved into ethanol absolute to act as the control group. The solution was irradiated by a 980 nm laser (1.5 W/cm^2^), and the absorbance of DPBF at 410 nm was recorded every 5 min in darkness.

### Cytotoxicity Assay

CCK8 (Cell counting kit-8, Meilunbio, Dalian) was applied for validating the cellular viability of UCNP@SiO_2_/MB@QCh. At first, the nanoparticles were dispersed in a DMEM culture medium at the concentration of 25, 50, 125, and 250 μg/ml. Meanwhile, mouse fibroblast L929 cells were seeded in 96-well plates at a density of 6 × 10^3^ cells per well for 2 days at 37°C (Sun et al., 2021). The control group contained 100 ml DMEM without UCNP@SiO_2_/MB@QCh. Then, 50 ml UCNP@SiO_2_/MB@QCh and 50 μl DMEM were added to cell plates, which means the ultimate concentrations of materials were 12.5, 25, 62.5, and 125 μg/ml. Then, the particles were incubated for 12, 24, and 36 h. After performing the abovementioned procedures, the residual liquid was removed from the wells and 100 μl fresh medium with 10 μl CCK-8 reagent solution was added to each well of the plate. Finally, OD450 was measured using a microplate reader. The relative cell viability was calculated by the following formula (Ji et al., 2021):


$$\begin{array}{l} \text{Viability }\left(\text{\%}\right)\;=\;{\text{OD}}_{\text{Test}}/{\text{OD}}_{\text{control}}\;\times \;100\% \end{array}$$

### *In vitro* Antibacterial Tests

*E. faecalis* ATCC 29212 was selected as the standard species of Gram-positive bacteria. *E. faecalis* strain was incubated in a brain heart infusion (BHI) medium, resulting in a density of 1 × 10^7^ colony-forming units (CFU) per ml. To avoid the heating effect of 980 nm laser irradiation, every cell of the 96-well plates containing 200 μl BHI with *E. faecalis* (1 × 10^7^ CFU/ml) was explored with NIR laser (1.5 W/cm^2^) for 10, 20, and 30 min, respectively. Furthermore, each well was irradiated for 1 min at an interval of 2 min (Zhang et al., 2018a; Li et al., 2019). Then, 20 μl suspension was taken out from each well, diluted with sterilized PBS, dropped on BHI agar plates, and incubated at 37°C for 24 h. The bacteria colony numbers of each group were calculated.

To select the appropriate irradiation time, 1.5 ml UCNP@SiO_2_/MB@QCh (2 mg/ml) and 1.5 ml bacterial solutions (1 × 10^7^ CFU/mL) were mixed and co-cultured for 1 h in a shaking incubator. Then, 200 μl suspension was added to a 96-well plate and irradiated by a 980-nm laser for 10, 20, and 30 min, respectively. 1.5 ml bacterial suspension with 1.5 ml BHI medium was set as the control group without NIR irradiation. However, the procedure was the same as that mentioned in the above experiment.

To investigate the antibacterial effect of UCNP@SiO_2_/MB and UCNP@SiO_2_/MB@QCh, 1 ml bacterial suspension was added to 1 ml of the above materials at varying concentrations (25, 50, 125, and 250 μg/ml), respectively. Then, the mixture was incubated in a shaking incubator for an hour (Li et al., 2017). Next, 200 μl mixture suspension was illuminated by a 980 nm laser for 10 min (Liu et al., 2020). The control group included the solution of 1 ml bacterial strain and 1 ml BHI medium. The steps that were followed were the same as before. Finally, the survival rates of *E. faecalis* were figured out by the following equation:


$$\begin{array}{l} \text{The relative survival rates}={\text{N}}_{\text{Text}}/{\text{N}}_{\text{Control}}\times 100\% \end{array}$$

Where N_Text_ means the colony number of the experimental group, and N_Control_ means the colony number of the control group_._

### Biofilm Inhibition Tests

First, the circle microscope cover glasses were placed in 75% alcohol for ultrasonic treatment for 30 min and then soaked for 8 h for disinfection. The drying surface liquid and the glasses were placed on a 12-well plate. Of note, 1 ml of *E. faecalis* suspension was added to each well and mixed properly, and the plate was placed in the incubator for 2 days. Then, the original medium was taken out. Meanwhile, 1 ml of fresh BHI medium was added to each well. In the control group, 1 ml of medium was added again, and in the experimental group, 1 ml UCNP@SiO_2_/MB@QCh (250 μg/ml) and 1 ml of CHX (2%) were added for co-culture for 1 h. Next, the wells with UCNP@SiO_2_/MB@QCh were exploded to 980 nm NIR irradiation for 10 min. Then, the liquid from each well was removed. Then, 2.25 μl PI and STYO9 were dissolved in 1.5 ml sterile DDW. Every cell-containing glass was injected with 200 μl of the mixture of PI and STYO9, and stained for 15 min. The solution was taken out and each glass was washed with PBS 3 times. Finally, fluorescence images from the stained bacteria were observed under a confocal laser scanning microscope (CLSM, Leica TCS SP8, Germany). Bacterial cell viability was determined by using propidium iodide (PI) and STYO9 staining dye (Zhu et al., 2020). Live bacteria were stained into green fluorescence and dead bacteria were stained into red fluorescence.

To observe the morphology of *E. faecalis*, the dentin plates from the third molar were adopted to culture *E. faecalis* strain. The plates were added to a 12-well plate and immerged in a 2-ml bacterial suspension. The culture lasted for 7 days, and the fresh culture BHI medium was substituted every 2 days. Then, 2 ml BHI medium with UCNP@SiO_2_/MB@QCh (125 μg/ml) was added into three wells, while the control group was added in 2 ml BHI. After 1 h, the experimental group was exploded to 980 nm NIR irradiation for 10 min. After glutaraldehyde fixation and alcohol gradient dilution, the samples were tested by SEM.

### Statistical Analysis

All data were shown as mean ± standard deviation (SD). All statistical analyses were evaluated using Graphpad Prism 8 or Origin 9. One-way analysis of variance (ANOVA) with Tukey's test was used to determine the differences between the groups. A value of *p* < 0.05 was considered to be statistically significant.

## Results and Discussion

### Physicochemical Characterization of UCNP@SiO_2_/MB@QCh

**Figure 1:**
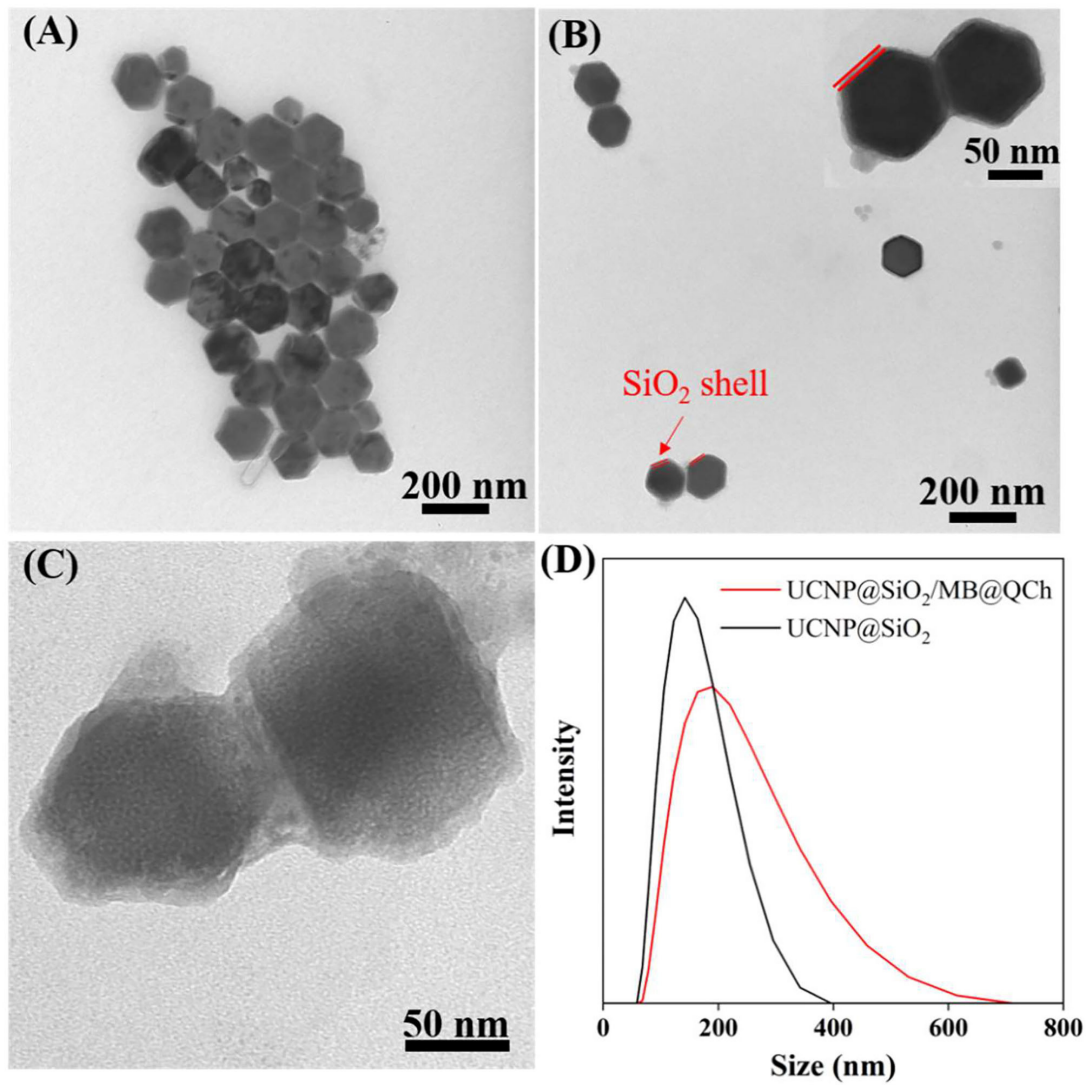
TEM images of **(A)** UCNP, **(B)** UCNP@SiO_2_, and **(C)** UCNP@SiO_2_/MB@QCh. **(D)** Diameter distributions of UCNP@SiO_2_ and UCNP@SiO_2_/MB@QCh.

UCNP@SiO_2_/MB and QCh were prepared by reported procedures (Liu et al., 2015; You et al., 2016). Then, UCNP@SiO_2_/MB@QCh was synthesized utilizing their electrostatic interaction, forming core-shell structured lanthanide-doped upconversion nanoparticles (Yin et al., 2016). To validate the core-shell structure of UCNP@SiO_2_/MB@QCh, TEM was employed to detect their structure at the nanoscale level. As shown in Figure 1A, the nanosized UCNP (~98 nm) presented the form of a pure hexagonal phase and dispersed homogeneously. Native UCNP showed low fluorescence resonance energy transfer (FRET) efficiency and considerable background, which may reduce the assay sensitivities (Li et al., 2015). To enhance the efficiency of FRET, as shown in Figure 1B, the dense silica layer with a thickness of 8 nm was smoothly wrapped around the UCNPs. After incorporation with QCh, the surface of UCNP@SiO_2_/MB@QCh became rough and its size increased to around 109 nm (Figure 1C). Besides, a double-layer around the core was identified, that is, the layer next to the core was the silica layer and the outermost layer was QCh (Lv et al., 2017). Furthermore, the particle size of UCNP@SiO_2_/MB@QCh in the aqueous solution detected by DLS was 187 nm, while the particle size of UCNP@SiO_2_ was 137 nm (Figure 1D). It may be concluded that the conjunction of QCh to UCNP@SiO_2_ was successfully achieved by the comparison of diameters between UCNP@SiO_2_ and UCNP@SiO_2_/MB@QCh. It was reported that when the nanomaterial size is smaller than 500 nm, they could better penetrate and destroy bacterial biofilms (Wu et al., 2021). The size of the prepared UCNP@SiO_2_/MB@QCh was much smaller than 500 nm, which indicates that it might enter inside bacterial films and then eradicate pathogenic bacteria.

**Figure 2:**
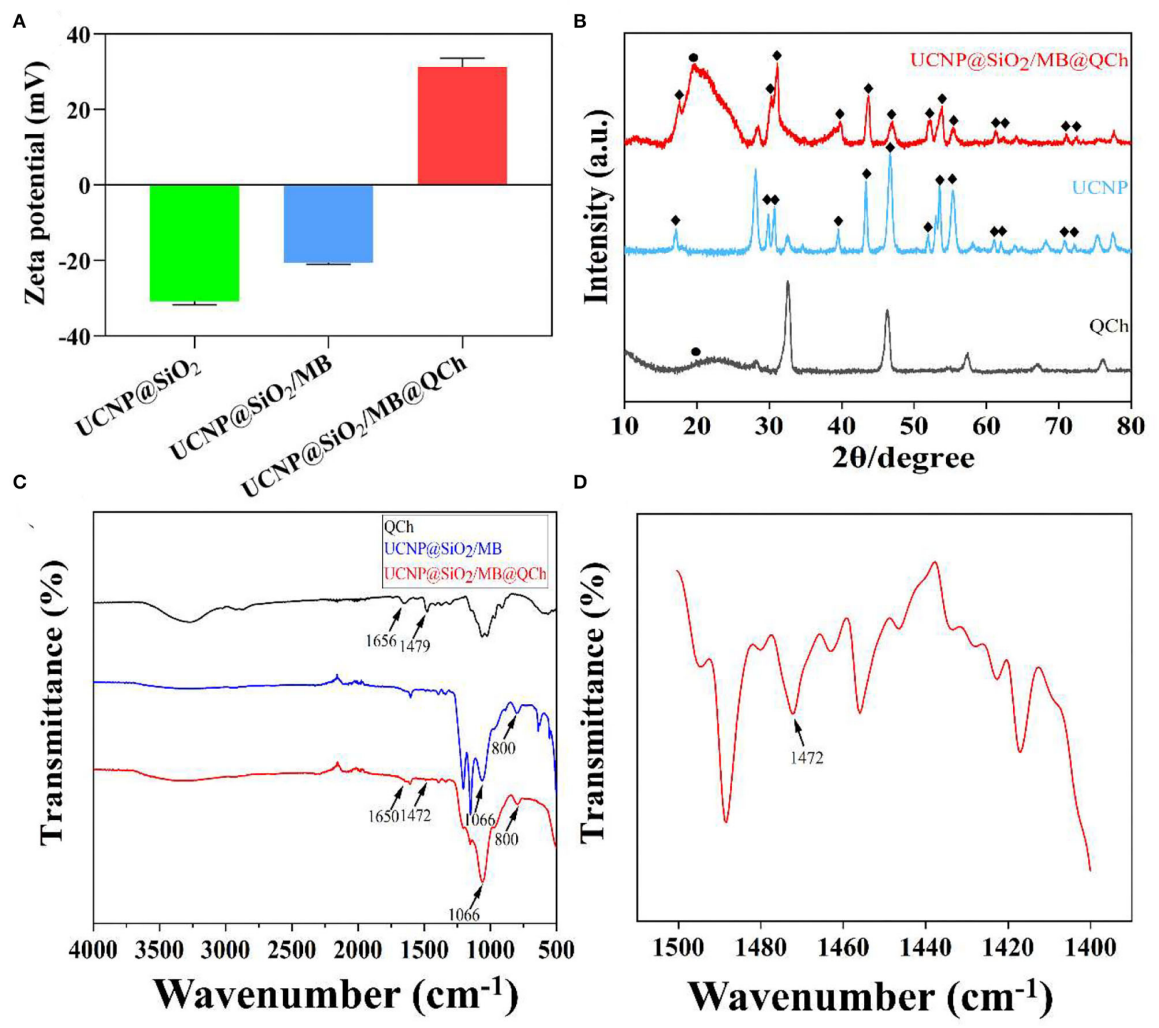
**(A)** Zeta potentials of UCNPs@SiO_2_-based nanoparticles. **(B)** XRD patterns of QCh, UCNP, and UCNP@SiO_2_/MB@QCh. **(C)** FTIR spectra of QCh and UCNPs@SiO_2_-based nanoparticles. **(D)** The local enlarged FTIR spectrum of UCNP@SiO_2_/MB@QCh between 1,400 and 1,500 cm^−1^.

It has been reported that nanoparticles with positive charges could interact and disturb the membrane of bacteria, which might increase the membrane permeability and lead them to death (Bing et al., 2016). Hence, we tested the charges of UCNP@SiO_2_, UCNP@SiO_2_/MB, and UCNP@SiO_2_/MB@QCh. As shown in Figure 2A, it was found that the zeta potential of UCNP@SiO_2_ was −30.8 mV, while that of UCNP@SiO_2_/MB was −20.2 mV. Conversely, the zeta potential of UCNP@SiO_2_/MB@QCh changed to 28.9 mV owing to the modification of positively charged QCh, which also reveals the successful construction of UCNP@SiO_2_/MB@QCh (Qiao et al., 2020). Choudhary and co-workers reported that nanofluids were regarded as moderately stable when their zeta potential was near ±30 mV(Choudhary et al., 2017).

**Figure 3:**
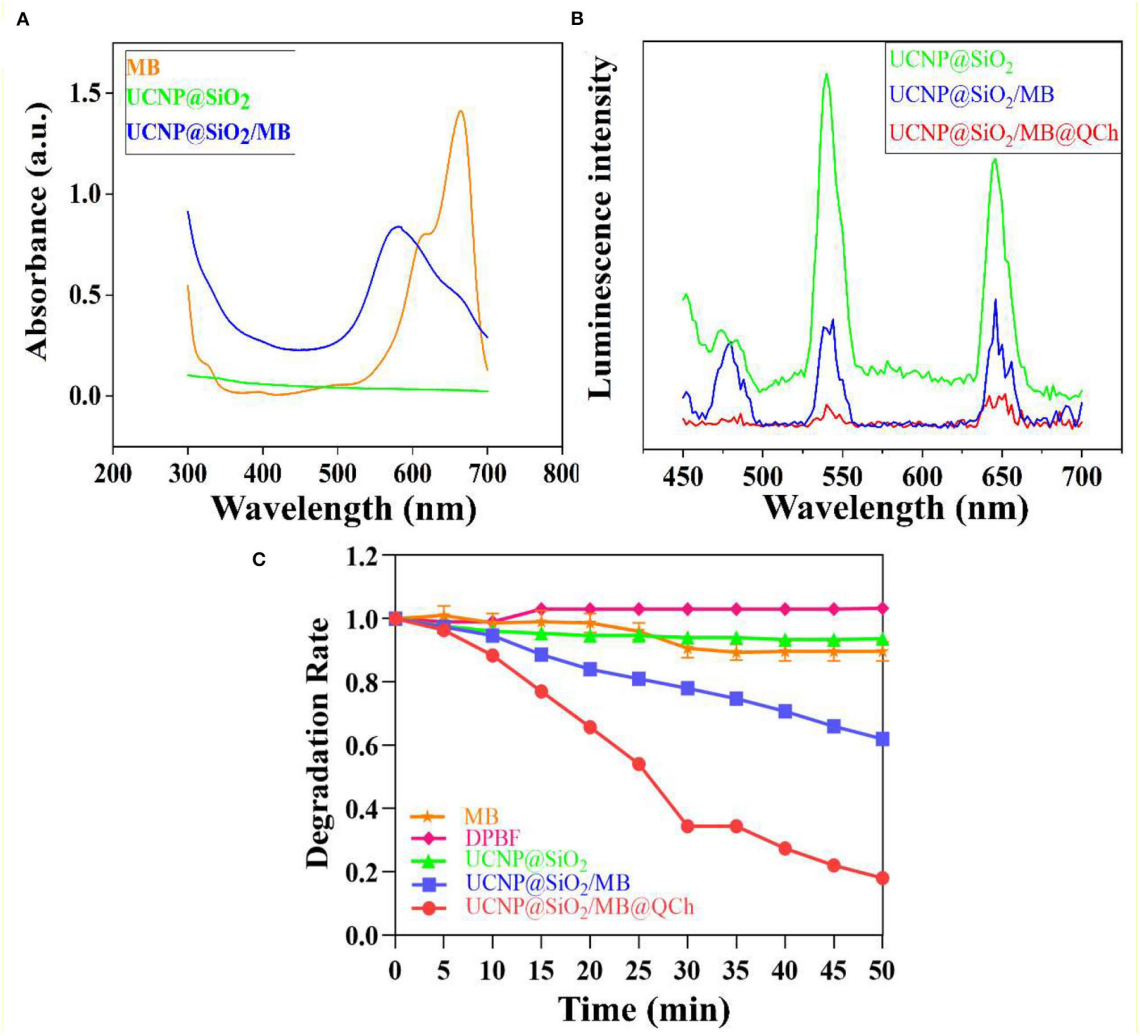
**(A)** The Uv-Vis spectra of MB, UCNPs@SiO_2_, and UCNPs@SiO_2_/MB. **(B)** The relative UCL efficiency of UCNPs@SiO_2_-based nanoparticles. **(C)** ROS detection of MB, DPBF, and UCNPs@SiO_2_-based nanoparticles in darkness.

Additionally, XRD patterns of UCNP and UCNP@SiO_2_/MB@QCh indicated that the prepared nanoparticles were in the form of a hexagonal phase (Figure 2B). The chemical feature of QCh, UCNP@SiO_2_/MB, and UCNP@SiO_2_/MB@QCh was measured by FTIR analysis (Figures 2C,D). After salinization, UCNP@SiO_2_/MB showed two strong absorption bands at 1,066 and 800 cm^−1^ due to asymmetric, symmetric stretching vibrations and bending vibrations of the Si-O-Si bond (Liu et al., 2020). This provided an elucidation for the successful synthesis of UCNP@SiO_2_/MB. In addition, the special sharp peak of QCh was observed at 1,479 cm^−1^, which corresponded to the methyl groups of ammonium (Song et al., 2010; You et al., 2016). It was also obtained at 1,472 cm^−1^ in UCNP@SiO_2_/MB@QCh. Meanwhile, the peak of C=O in QCh appeared at 1,656 cm^−1^, which was detected at 1,650 cm^−1^ on the latter (Niamlang et al., 2017). These results imply that QCh was successfully coated onto UCNP@SiO_2_/MB. Furthermore, as shown in Figure 3A, the UV-Vis spectra of MB, UCNPs@SiO_2_, and UCNPs@SiO_2_/MB indicated that MB was successfully loaded into the SiO_2_ layer (Yue et al., 2018).

### Luminescence Ability and ROS Formation Induced by UCNP@SiO_2_/MB@QCh

To confirm the luminescence ability of the samples, we tested the relative luminescence intensity of UCNPs@SiO_2_-based nanoparticles. Findings revealed that the strong absorption peak of MB was 654 nm and the emission wavelength of UCNP was 650 nm, which matched well (Yin et al., 2014). Therefore, the FRET between MB and UCNPs may exist with high efficiency to further generate increased ROS to eradicate pathogenic bacteria. Figure 3B shows that the luminescence intensity at 650 nm of UCNP@SiO_2_/MB was reduced by 64% compared with UCNPs@SiO_2_. UCNP@SiO_2_/MB@QCh decreased by 88%. Interestingly, QCh may contribute to enhancing the FRET efficiency of the nanoparticles.

The antimicrobial application of UCNP@SiO_2_/MB@QCh mainly depends on its ability to produce ROS species, especially singlet oxygen (^1^O_2_) (Wang et al., 2020b; Zhu et al., 2022). Due to the greater penetration of NIR laser compared with visible light, UCNP@SiO_2_/MB@QCh could be applied for antimicrobial activity in deeper tissues, such as apical periodontium. In our work, UCNP could activate MB to product ^1^O_2_ to react with DPBF. This could generate uncolored 1,2-dibenzoylbenzene (DBB) as the product (Qi et al., 2019; Zhao et al., 2020). Subsequently, the absorption value of DPBF at 410 nm would decrease with the generation of DBB. As shown in Figure 3C, the degradation rate of MB or UCNP@SiO_2_ alone dropped a little after irradiation of 980 nm NIR, while that of DPBF did not change. However, the degradation rate of UCNP@SiO_2_/MB@QCh significantly decreased by 82% at 50 min, while that of UCNP@SiO_2_/MB reduced by 38%. Besides, the degradation rates decreased gradually with the extension of NIR time. Therefore, we may conclude that compared with UCNP@SiO_2_/MB, UCNP@SiO_2_/MB@QCh produced a significant amount of ^1^O_2_ and could possess a higher photodynamic ability, which was supposed to play an important role to eliminate bacteria. It has been reported that QCh may increase the conversion efficiency of UCNP to generate more ROS (Liao et al., 2019). Another striking example reported by Hu and coworkers showed the crucial effect of ROS to irreversibly destroy the membrane structure of bacteria, thus leading to the death of bacteria (Hu et al., 2019).

### Cytocompatibility and Antibacterial Assessment of UCNP@SiO_2_/MB@QCh

**Figure 4:**
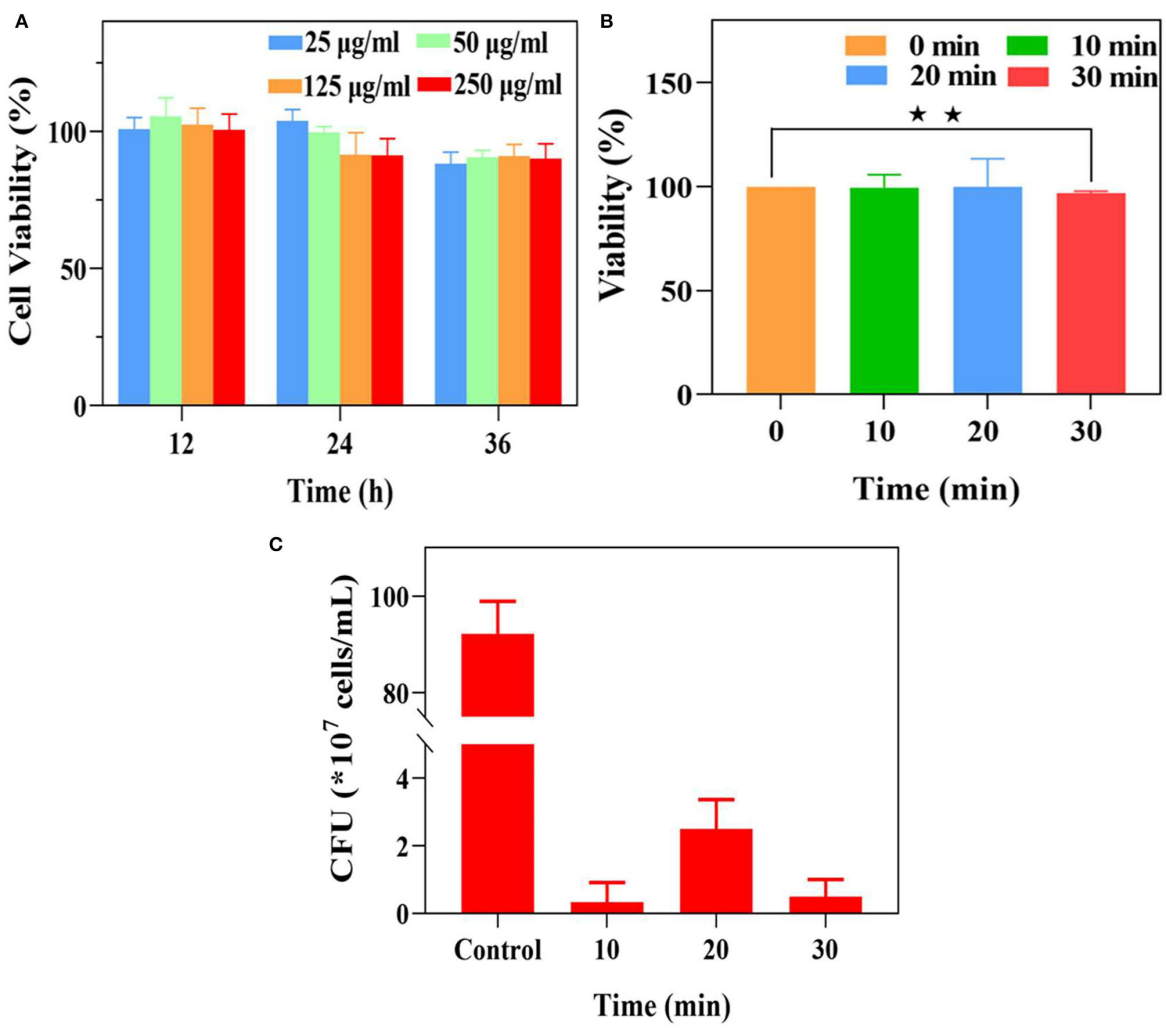
**(A)** The cell viability of L929 fibroblasts with different concentrations of UCNP@SiO_2_/MB@QCh for 12, 24, and 36 h. **(B)** The viability of *E. faecalis* after different irradiation times. **(C)** The residual CFU of *E. faecalis* was treated with UCNP@SiO_2_/MB@QCh after different irradiation times. *p < 0.05, **p < 0.01.

The potential cytotoxicity of nanoparticles is a crucial indicator in biomedical applications. Thus, the cytotoxicity assay was performed on L929 fibroblasts in our work (Sun et al., 2019; Wu et al., 2019). As demonstrated in the result of the CCK-8 assay (Figure 4A), the cell viabilities of L929 fibroblasts with UCNP@SiO_2_/MB@QCh in different co-culture times were all above 90%, indicating that the toxicity of nanoparticles toward cells might not significantly increase with time. In addition, after incubation with different concentrations of UCNP@SiO_2_/MB@QCh, the cell viabilities of L929 fibroblasts were nearly 90%, which suggests excellent cytocompatibility of the developed nanoparticles.

Each well of *E. faecalis* was irradiated for 1 min and at an interval of 2 min to avoid the heating effect of NIR light. As shown in Figure 4B, the vitality of *E. faecalis* significantly decreased when the irradiation time was 30 min, while their changes were negligible at 10 and 20 min. It could be considered that NIR irradiation at 10 and 20 min could not reduce the vitality of *E. faecalis*. Furthermore, to identify the proper irradiation time, the mixture of *E. faecalis* and UCNP@SiO_2_/MB@QCh was explored using a 980 nm laser for 10, 20, and 30 min, respectively. The vitalities of *E. faecalis* were significantly declined when irradiated for more than 10 min. Therefore, the irradiation time of 10 min was selected in the following experiment (Figure 4C).

**Figure 5:**
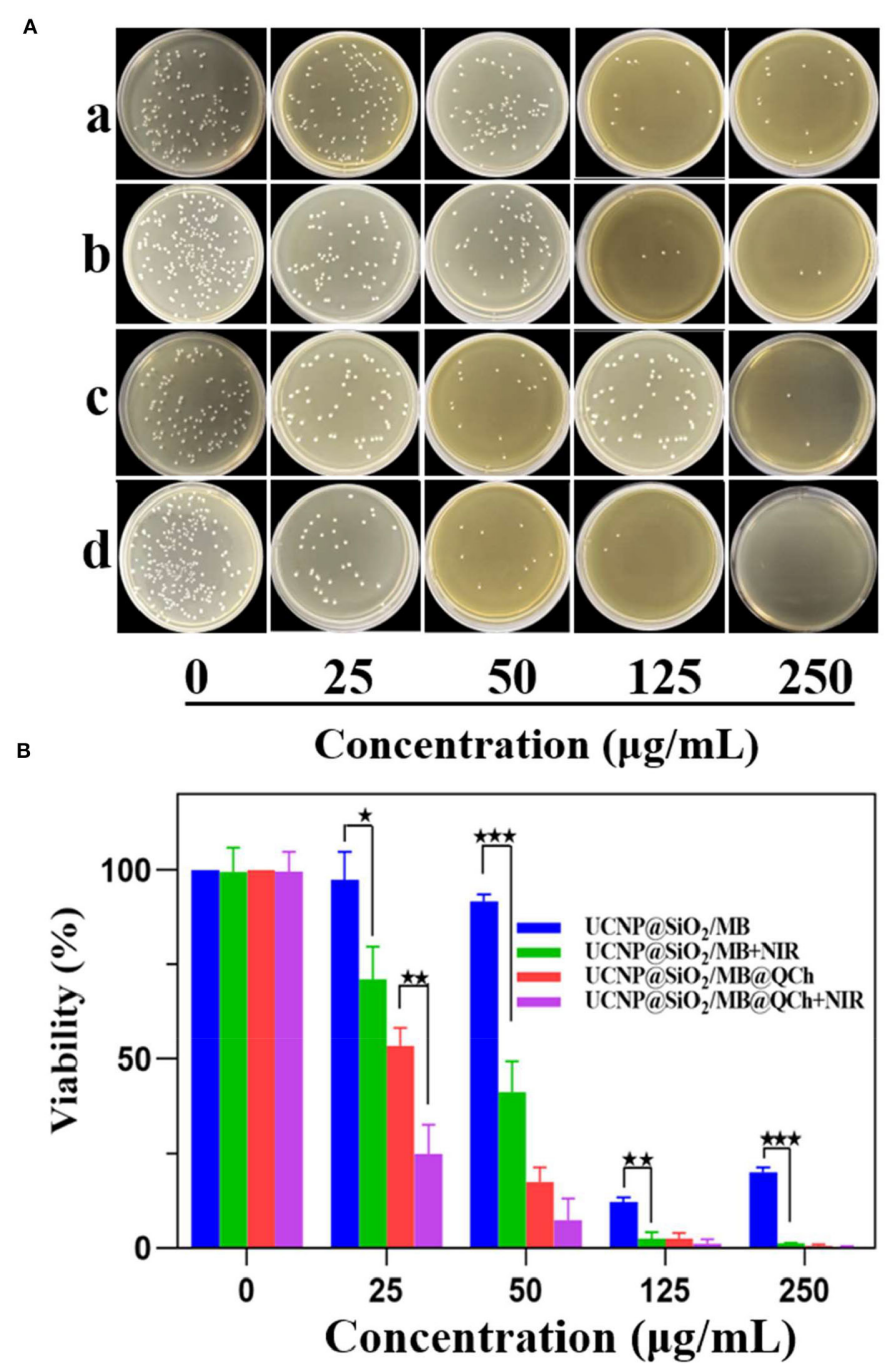
**(A)** Images of residual bacterial colonies of *E. faecalis* on agar plates after incubation with different concentrations of UCNP@SiO_2_/MB and UCNP@SiO_2_/MB@QCh, followed by further treatment with and without NIR irradiation (980 nm, 1.5 W cm^−1^). (a) means UCNP@SiO_2_/MB without NIR, (b) means UCNP@SiO_2_/MB with NIR, (c) means UCNP@SiO_2_/MB@QCh without NIR, and (d) means UCNP@SiO_2_/MB@QCh with NIR. **(B)** Calculated survival ratio of *E. faecalis* after corresponding treatments. *p < 0.05, **p < 0.01, ***p < 0.001.

To investigate the antibacterial effect of UCNP@SiO_2_/MB@QCh, *E. faecalis* was incubated with the nanomaterials for 1 h and irradiated by 980 nm NIR for 10 min (Lv et al., 2022). We found that UCNP@SiO_2_/MB@QCh displayed a better antimicrobial effect than UCNP@SiO_2_/MB with or without NIR laser (Figures 5A,B). As shown in Figures 5A,B, the survival rates subsequently decreased with the increasing concentration of UCNP@SiO_2_/MB@QCh. When the concentration arrived at 250 μg/ml, the survival rate of *E. faecalis* reduced to 0.6% without NIR irradiation. Meanwhile, it decreased to 0.2% after NIR irradiation, revealing that ROS could further lead *E. faecalis* to death. The reason may be that UCNPs were excited by NIR irradiation, which made MB generate ROS to kill bacteria (Wang et al., 2021a; Wu et al., 2022). Giannelli and co-workers confirmed that MB can produce single oxygen under specific conditions, which may generate hydroxyl radicals(OH–) and other kinds of ROS to remove bacteria (Giannelli and Bani, 2018). Due to the coating of QCh, UCNP@SiO_2_/MB@QCh exhibited positive charges. Hence, it may identify, adhere to, and destroy *E. faecalis* more efficiently. Cheah et al. reported that chitosan and its derivatives disrupt bacteria integrity and the intracellular components are leaked (Cheah et al., 2019). Therefore, UCNP@SiO_2_/MB@QCh obtained a better antibacterial effect than UCNP@SiO_2_/MB. UCNP@SiO_2_/MB@QCh performed an excellent inhibiting effect against *E. faecalis*, showing a promising application prospect.

### Biofilm Inhibition Evaluation of UCNP@SiO_2_/MB@QCh

**Figure 6:**
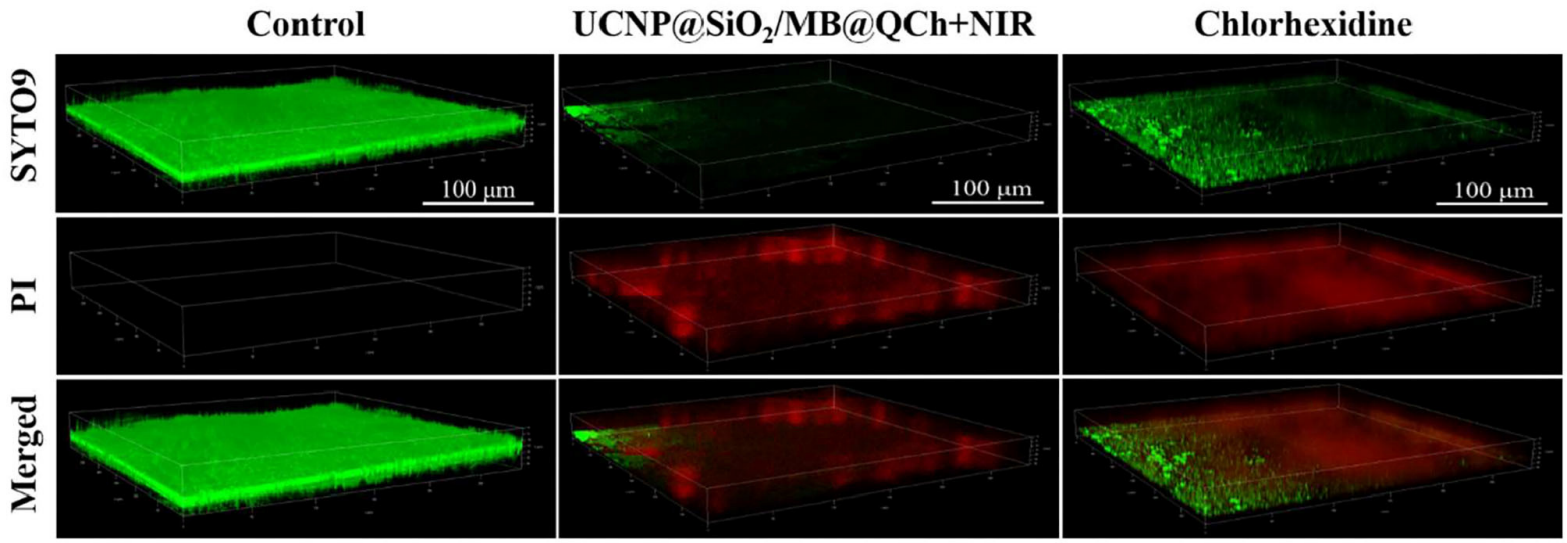
3D-CLSM images of *E. faecalis* biofilms in control, UCNP@SiO_2_/MB@QCh, and chlorhexidine groups. Green (SYTO9) and red (PI) represent the live and dead cells of *E. faecalis*, respectively.

*E. faecalis* biofilm is a key primary cause of PEIs (El karim et al., 2007; Keskin et al., 2017). To further verify the antibiofilm properties of UCNP@SiO_2_/MB@QCh, we conducted antibacterial experiments on the biofilm of *E. faecalis*. As shown in Figure 6, a uniform green *E. faecalis* biofilm was detected in the control group, which demonstrated that a uniform biofilm model was established (Qi et al., 2021). In the experimental group, after being treated with UCNP@SiO_2_/MB@QCh, the area of green fluorescence narrowed down, and the red fluorescence showed higher intensity and wider range, which indicates that its biofilm structure was loose, and its biofilm thickness decreased. Importantly, the anti-biofilm ability of UCNP@SiO_2_/MB@QCh is comparable to commercial chlorhexidine.

**Figure 7:**
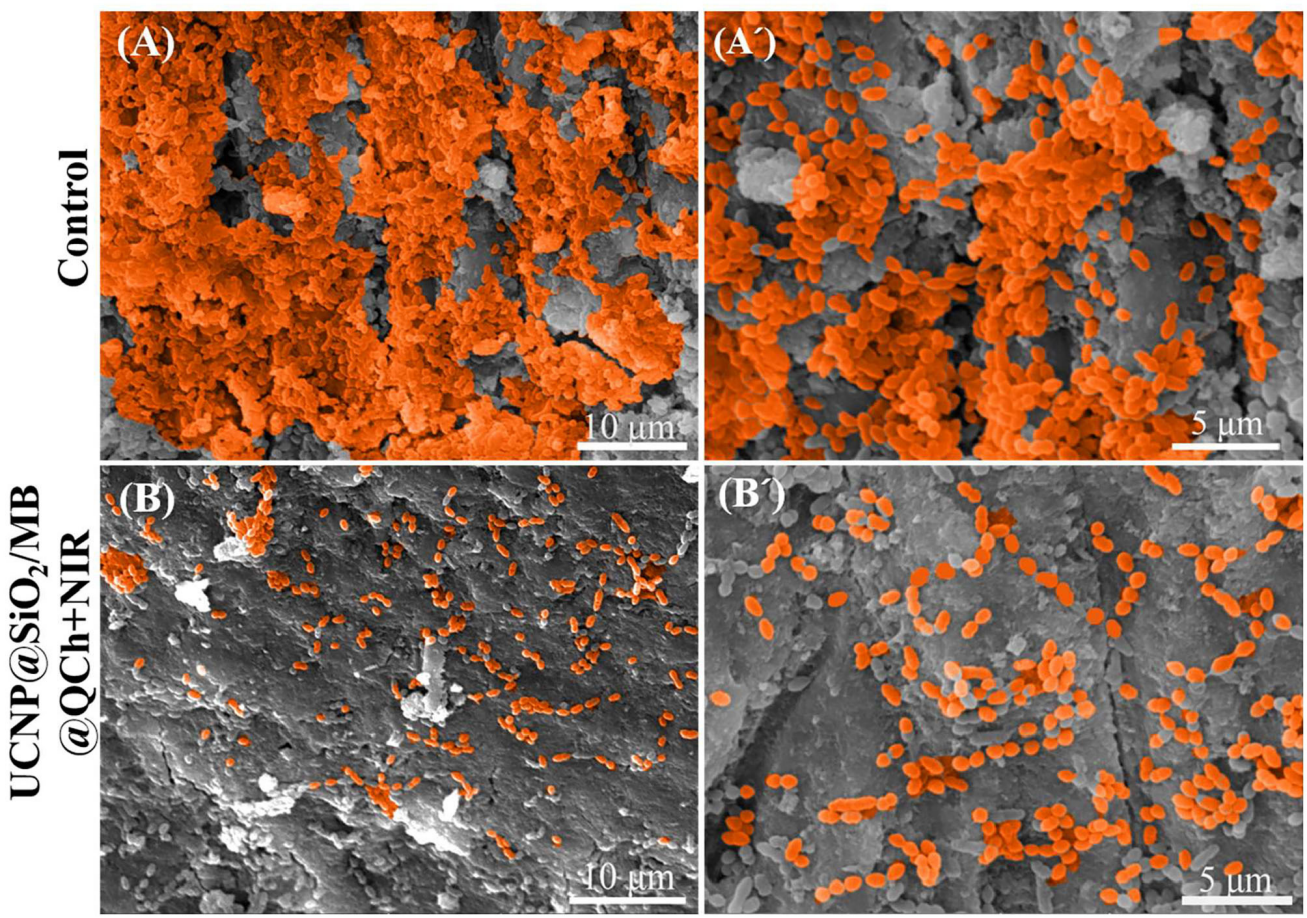
SEM images of *E. faecalis* **(A,A')** without and **(B,B')** with the treatment of UCNP@SiO_2_/MB@QCh.

As shown in Figure 7, SEM was employed to detect the effect of UCNP@SiO_2_/MB@QCh on *E. faecalis* biofilm on the root canal surface. It was found that the amount of *E. faecalis* in the UCNP@SiO_2_/MB@QCh group was greatly decreased compared with that of the control. The *E. faecalis* biofilm in the UCNP@SiO_2_/MB@QCh group was destroyed, and its bacteria were sparsely dispersed. QCh could adsorb bacteria and then destroy their cell membranes, resulting in the efflux of DNA and RNA from the cytoplasm, thus achieving the effect of killing bacteria (Abd El-Hack et al., 2020). Combined with the action of ROS, UCNP@SiO_2_/MB@QCh achieved an antibacterial double impact.

## Conclusion

UCNP@SiO_2_/MB@QCh with improved biocompatibility and enhanced antibacterial performances were successfully fabricated for the management of PEIs. The size of UCNP@SiO_2_/MB@QCh (187 nm) was significantly larger than that of UCNP@SiO_2_ (137 nm) due to the coating of QCh. In the course of aPDT treatment, more than 99% of *E. faecalis* were eliminated by the UCNP@SiO_2_/MB@QCh. Furthermore, accompanied by the modification of positive charge QCh, the germicidal efficiency was remarkably higher than that of UCNP@SiO_2_/MB. Such excellent antibacterial performance benefited from the close attachment with bacteria caused by QCh. Also, as a proverbial biocompatible and non-toxic material and widely used in biological fields, the addition of QCh greatly reduced the toxicity of aPDT photosensitizer and improved its biosafety. Thus, UCNP@SiO_2_/MB@QCh provides a novel strategy to eradicate PEI-associated bacteria for potential practical applications.

## Data Availability Statement

The original contributions presented in the study are included in the article/supplementary material, further inquiries can be directed to the corresponding authors.

## Author Contributions

BZ and XL carried out the experiments, performed data analysis, and wrote the manuscript. QX designed the experiments, analyzed the results, and wrote the manuscript. PG analyzed the results and wrote the manuscript. QZ designed the experiments, revised the manuscript, and did project administration. All authors read and approved the final manuscript.

## Funding

The authors are very thankful for financial support from the National Natural Science Foundation of China (Grant No. 31900957), the Shandong Provincial Natural Science Foundation (Grant No. ZR2019QC007), the Innovation and Technology Program for Excellent Youth Scholars of Higher Education of Shandong province (Grant No. 2019KJE015), the Traditional Chinese Medicine Science and Technology Project of Shandong province (Grant No. 2021Q069), and the Natural Science Foundation of Shandong Province (ZR2020MH183).

## Conflict of Interest

The authors declare that the research was conducted in the absence of any commercial or financial relationships that could be construed as a potential conflict of interest.

## Publisher's Note

All claims expressed in this article are solely those of the authors and do not necessarily represent those of their affiliated organizations, or those of the publisher, the editors and the reviewers. Any product that may be evaluated in this article, or claim that may be made by its manufacturer, is not guaranteed or endorsed by the publisher.
